# Impact of MRI on target volume definition in head and neck cancer patients

**DOI:** 10.1186/s13014-023-02326-0

**Published:** 2023-09-06

**Authors:** Kerstin Clasen, Marcel Nachbar, Sergios Gatidis, Daniel Zips, Daniela Thorwarth, Stefan Welz

**Affiliations:** 1 Department of Radiation Oncology, University Hospital Tübingen, University of Tübingen, Tübingen, Germany; 2 Section for Biomedical Physics, Department of Radiation Oncology, University Hospital Tübingen, University of Tübingen, Tübingen, Germany; 3 Department of Radiology, University Hospital Tübingen, University of Tübingen, Tübingen, Germany; 4 Department of Radiation Oncology, Marienhospital, Stuttgart, Germany

**Keywords:** Radiotherapy, IMRT, MRI imaging, Local control, Target volume delineation, HNSCC

## Abstract

**Background:**

Target volume definition for curative radiochemotherapy in head and neck cancer is crucial since the predominant recurrence pattern is local. Additional diagnostic imaging like MRI is increasingly used, yet it is usually hampered by different patient positioning compared to radiotherapy. In this study, we investigated the impact of diagnostic MRI in treatment position for target volume delineation.

**Methods:**

We prospectively analyzed patients who were suitable and agreed to undergo an MRI in treatment position with immobilization devices prior to radiotherapy planning from 2017 to 2019. Target volume delineation for the primary tumor was first performed using all available information except for the MRI and subsequently with additional consideration of the co-registered MRI. The derived volumes were compared by subjective visual judgment and by quantitative mathematical methods.

**Results:**

Sixteen patients were included and underwent the planning CT, MRI and subsequent definitive radiochemotherapy. In 69% of the patients, there were visually relevant changes to the gross tumor volume (GTV) by use of the MRI. In 44%, the GTV_MRI would not have been covered completely by the planning target volume (PTV) of the CT-only contour. Yet, median Hausdorff und DSI values did not reflect these differences. The 3-year local control rate was 94%.

**Conclusions:**

Adding a diagnostic MRI in RT treatment position is feasible and results in relevant changes in target volumes in the majority of patients.

## Background

In patients with locally advanced squamous cell carcinomas of the head and neck (LAHNSCC), definitive radiochemotherapy is a standard treatment option [1, 2]. However, locoregional failure is a major issue in these patients as locoregional control rates after two years were reported to be merely about 63% in a multicenter study of the German Cancer Consortium Radiation Oncology Group [3]. Therefore, accurate definition of the macroscopic tumor is of crucial importance, especially since the predominant pattern of failure in locally advanced head and neck squamous cell carcinoma (LAHNSCC) is local [4].

Magnetic resonance imaging (MRI) can add important information for radiotherapy planning as the soft-tissue contrast is enhanced compared to computed tomography (CT) imaging and additional image information can be obtained e.g. by functional MRI [5, 6].Therefore, a consensus guideline for the use of offline-MRI aided treatment planning was published in 2016 [7]. According to these recommendations, MRI imaging should be performed in treatment position using immobilization devices where suitable [7].

In our pilot study, we investigated the feasibility and the impact of diagnostic MR imaging in treatment position for radiotherapy planning. We investigated a recent MRI technology (MAGNETOM Vida, 3T, Siemens Healthineers, Erlangen, Germany) to ensure up-to-date imaging quality.

The aim of this prospective study was to optimize target volume definition and to assess the impact of the additional use of the MRI information on the delineation of the primary tumor.

## Methods

Among patients with LAHNSCC who were treated with primary, curatively intended radiochemotherapy, we prospectively investigated the subset of patients who received (in addition to the standard diagnostic CT scan) a diagnostic MRI (MAGNETOM Vida, 3T, Siemens Healthineers, Erlangen, Germany) in radiotherapy (RT) position prior to planning.

All patients declared their informed consent and the study was approved by the local ethics committee (445/2016BO2).

For every patient, a contrast enhanced CT of the whole body and the head and neck region was available. One patient received an additional FGD-PET-CT. After the planning CT (3 mm slices, thermoplastic individual mask, flat table, head and neck support, no contrast agent), patients underwent a diagnostic MRI with the same immobilization devices. The overall duration of the MRI sequences was about 25 min.

All MRI examinations were performed on a state-of-the-art 3 Tesla MRI scanner covering the anatomic region between skull base and upper thorax. The MRI protocol consisted of the following sequences: a transversal T2-weighted turbo spin echo (TSE) inversion recovery sequence, a transversal diffusion-weighted echo-planar imaging sequence with b-values of 50 and 800 mm^2^/s, a transversal dynamic contrast-enhanced (0.1 ml/kg Gadovist) T1-weighted gradient echo (GRE) sequence with a temporal resolution of 8 s and a contrast-enhanced 3D isotropic (1mm^3^) T1-weighted GRE sequence. All image volumes were corrected for distortion as part of the vendor-provided reconstruction workflow resulting in a geometrical error below pixel resolution for TSE and GRE sequences.

The MRI was then co-registered to the planning CT. A single radiation oncologist with 20 years of clinical experience and specializing in head and neck cancer did the contouring for all patients. A second physician provided assistance and acted as double verification. For the interpretation of the MRI images, advice from the colleagues of the department of diagnostic radiology was used when needed. We used lymph node size, shape, contrast enhancement and the location with respect to the primary tumor or adjacent lymph nodes to establish the likelihood for a node to be involved. Target volume delineation for the primary tumor was first done with all available diagnostic information but blinded for the MRI (contour GTV_CT). In a second step, the additional information from the MRI (i.e. T2 weighted imaging, contrast enhanced T1 imaging, diffusion weighted imaging, DWI) was taken into account and a new volume was derived that considered all former information plus the MRI (coregistered T2 and contrast enhanced T1 sequences) imaging (GTV_MRI). Radiotherapy planning was done using the GTV_MRI volume. If the MRI revealed additional suspect lymph nodes, this information was used for the definition of macroscopic tumor nodes but was not part of the investigation. As per institutional policy, high- and low risk elective volumes were delineated, and all target volumes were expanded for 6 mm craniocaudally and for 5 mm in every other direction to obtain a planning target volume (PTV). The prescribed doses were 70/60/54 Gy to the respective volumes using a volumetric modulated arc therapy (VMAT) technique. All patients received concurrent chemotherapy.

**Fig. 1:**
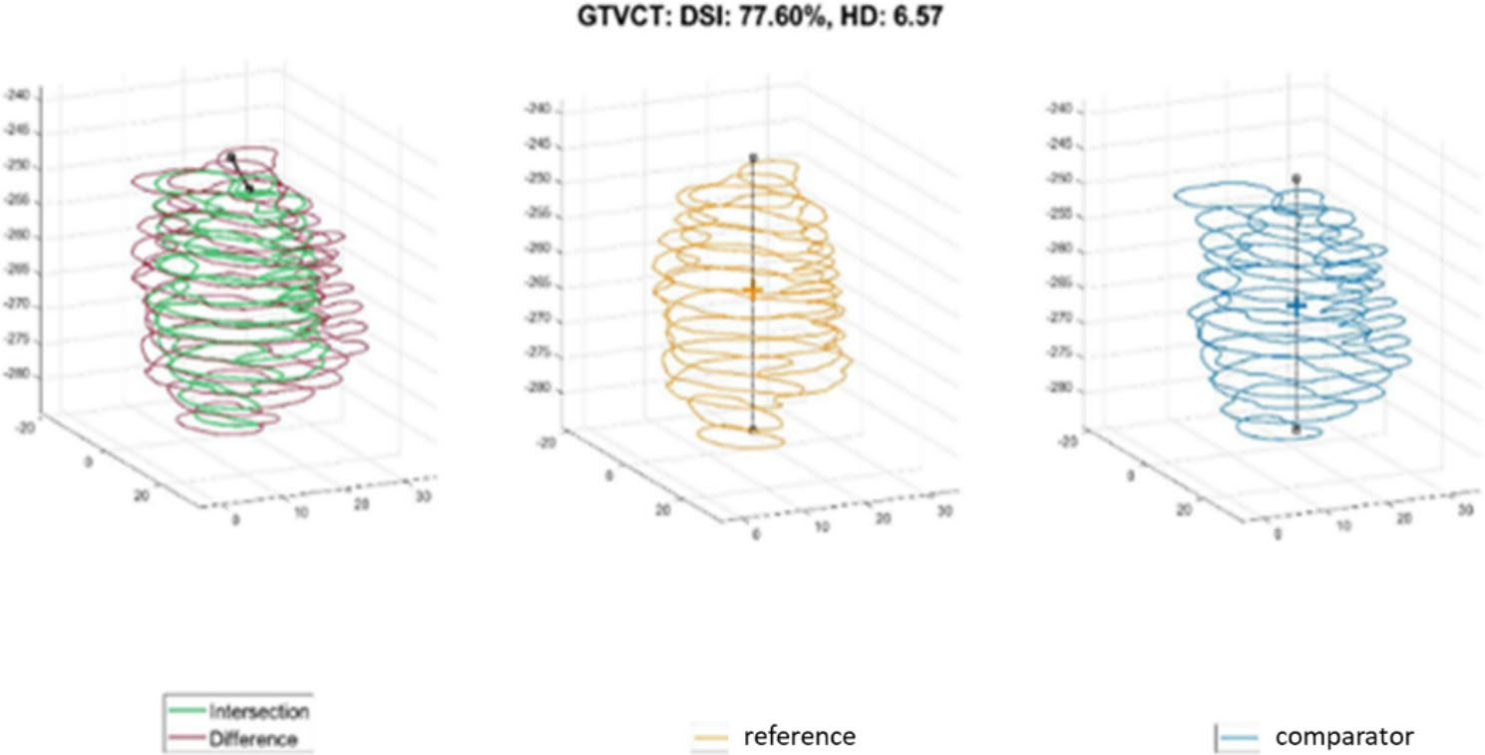
Example of the mathematical comparison between CT and MRI contour. Reference = CT, DSI = dice similarity index, HD = Hausdorff distance

We investigated the impact of the additional MRI information on the target volume for the primary tumor. This was done visually by assessing the difference between GTV_CT and GTV_MRI and respective scorings as relevant (i.e. if it was obvious, that the additional (MRI-derived) tumor volume would not have received a sufficient dose or that the omitted (CT-based) target volume would have caused additional toxicity) or as not relevant. Additionally, we used mathematical measures to describe the difference between the two target volumes. We used two established metrics to compare the CT-derived and MRI-derived target volumes (Example in Fig. 1).

1. HD95: This represents 95% of the Hausdorff distance which describes the maximum deviation of the volumes A and B in mm [$95HD=percentile({d}_{A,B} \cup {d}_{B,A},95th)$].
2. DSI: The dice similarity index describes the comparability of the volumes A and B in % [$DSC=\frac{2*|A\cap B|}{\left|A\right|+\left|B\right|}$]

## Results

From December 2017 to January 2019, 16 LAHNSCC patients received an additional MRI in RT position and were included in this study. The median age was 63 years (range: 52–76 years), 13 were male and 3 were female. In nine patients, p16 staining was positive, five patients were p16 negative and in two patients data about p16 is missing. Ten patients had a smoking history, four were non-smokers and in two patients the smoking status is unknown. Further patient characteristics are shown in Table 1. The median follow-up was 3 years.

**Table 1:** Patient characteristics. GTV = gross tumor volume

Group	(A) No relevant change	(B) Relevant change	(C) All patients
Number of patients	5	11	16
**Stage**			
T2	2 (40%)	2 (18%)	4 (25%)
T3	2 (40%)	4 (36%)	6 (38%)
T4	1 (20%)	5 (45%)	6 (38%)
**Grading**			
G2	2 (40%)	7 (64%)	9 (56%)
G3	2 (40%)	3 (27%)	5 (31%)
Unknown	1 (20%)	1 (9%)	2 (13%)
**Localization**			
Tonsil	3 (60%)	6 (55%)	9 (56%)
Base of tongue	2 (40%)	2 (18%)	4 (25%)
Hypopharynx	0 (0%)	2 (18%)	2 (13%)
Epipharynx	0 (0%)	1 (9%)	1 (6%)
**GTV (ml)** ***median (range)***			
CT	16.4 (6.4–28.4)	16,8 (2.8–173)	16.9 (2.8–173)
MRI	14.8 (7.4–27.7)	16.4 (3.3–183)	15.5 (3.3–183)
**Follow-up (years)** ***median (range)***	3 (2.5-3)	3 (0.5–3.5)	3 (0.5–3.5)

The median GTV of the primary tumor was 16.9 ml for the CT contour and 15.5 ml for the CT and MRI contour. There was one exceptional large tumor volume with 173/183 ml in CT/MRI, respectively, all other GTVs were in the range of 2.8–53.2/3.3–44.7 ml, respectively. Only one patient had an FDG-PET-CT as diagnostic procedure which did not change the CT or MRI volume.

The generated GTV based on CT and the additional volume with inclusion of MRI information were compared visually and deviations were rated as clinically relevant or not. Examples of target volume deviations between CT and MRI-based contours (relevant versus irrelevant changes) are visualized in Fig. 2a and b.

**Fig. 2a:**
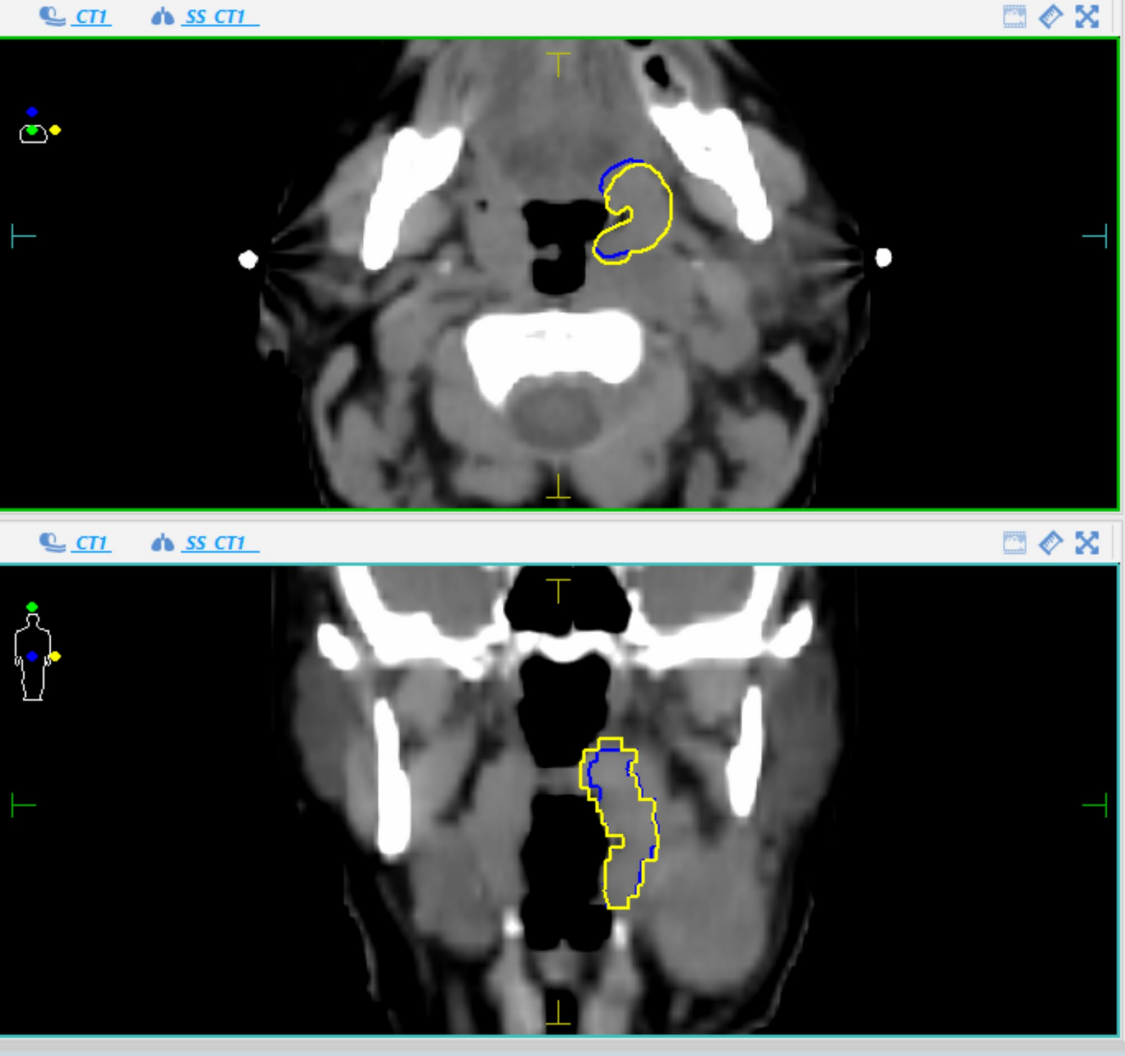
An example of “no relevant change”. Blue = CT contour, Yellow = MRI contour

**Fig. 2b:**
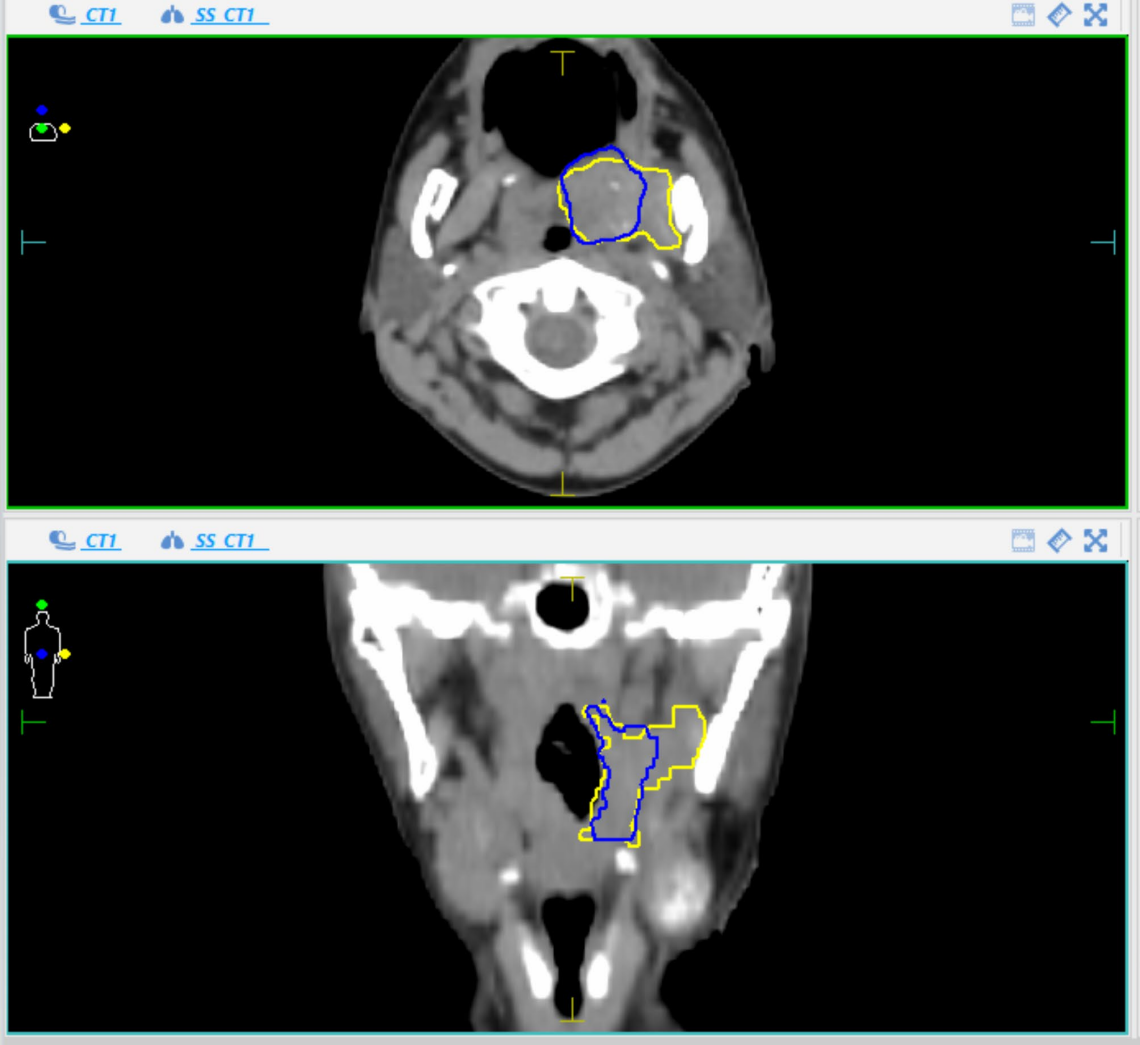
An example of “relevant change”. Blue = CT contour, Yellow = MRI contour

The quantitative comparison of the CT and MRI volumes is detailed in Table 2.

**Table 2:** Differences between CT and MRI delineation

Group	(A) No relevant change	(B) Relevant change	(C) All patients
Number of patients	5	11	16
Absolute volume (MRI-CT) [ml] (median; range)	-0.5 (-4.7–1)	4 (-8.5–10)	0.75 (-8.5–10)
Relative volume (MRI-CT) [%] [median, range]	-2.46 (-27.7–15.63)	19.64 (-18.4–47.78)	10.7 (-27.7–47.8)
DSI [median, range]	0.734 (0.606–0.919)	0.775 (0.507–0.942)	0.769 (0.507–0.942)
HD95 [mm] [median, range]	5.97 (2.92–9.06)	6.41 (2.82–19.21)	5.97 (2.82–19.21)
Final GTV not contained in PTV (CT)	0 (0%)	7 (64%)	7 (44%)

There were large differences of up to 47.8% with a median of 2.5% in patients without relevant changes and a median of 19.6% in patients with relevant changes. The DSI and HD95 values did not correlate with the visual judgement of changes which were judged relevant in 69% of all patients. In 44% of all patients, the final GTV (including the MRI information) would not have been covered by the PTV generated using the initial CT-based contour (with a margin of 5 and 6 mm).

The most frequently involved anatomical region concerning relevant changes was the tongue or the base of tongue and the soft palate or the lateral pharyngeal wall.

With the resulting delineation based on CT and MRI, three-year local control (LC) rate was 94%. The only local failure occurred in the patient with the exceptionally large tumor of 173/183 ml which extended in the maxillary sinus and the parapharyngeal space.

## Discussion

MRI achieves a better soft tissue contrast compared to CT [8] resulting in additional information for the contouring of the primary tumor in LAHNSCC. We showed that an MRI in radiotherapy treatment position, which is the best option considering the uncertainties with registration of an MRI in a different position [9, 10], is feasible and can be used for target volume definition.

The comparison of the GTV obtained by CT only versus CT and MRI revealed large differences. In this respect, the median DSI was only 77% and the mean HD95 was 6 mm. In 12 of the 16 patients, there was a difference of more than 10% between the two contouring approaches. Yet, when comparing the median values of the respective mathematical evaluations (Table 2), these differences are not reflected.

This is most likely caused by the fact that tumor volumes were extended due to MRI imaging in some directions but reduced in other directions within the same GTV, which cannot be adequately assessed by median values of a volume-based evaluation.

Therefore, we chose to primarily investigate resulting changes as relevant (i.e. clinically meaningful) or not by visual evaluation (Examples are depicted in Fig. 2a and b). In this respect, there were 7 patients whose larger final GTVs (GTV_MRI) exceeded even the PTV contour obtained based on the CT only and therefore would not have received a sufficient dose. In 4 patients, the GTVs became smaller.

There is scarce literature on MRI target volume definition in head and neck cancer. In nasopharyngeal carcinomas, investigations showed an improved definition of the primary tumor, e.g. with respect to the skull base [8]. In our study, the most frequent locations with relevant changes were anatomical regions where soft tissue contrast is crucial, like the tongue or the soft palate. A polish study [11] using PET/MRI found larger GTVs in 8 of 10 patients when using the MRI information in tongue carcinomas, corroborating our findings. In oropharyngeal carcinomas, Thiagarajan et al. found smaller MRI based volumes for the primary tumor compared to CT based contours [12]. This is in concordance with our findings, with deviations in both directions.

A group from New Delhi reported significant differences in GTV delineation with the use of MRI in 25 patients [13] as well as a british study [14] in tonsil and base of tongue carcinomas. The only study who concluded that CT and MRI based GTV volumes were similar used PET/MRI in 9 patients [15] and nevertheless found a DSC value of 0.63 and acknowledged individual cases with larger differences.

The local control rate of 94% after three years is high considering the frequency of 38% T4 and 38% T3 primaries and would have been 100% if one exceptionally large and atypical tumor would have been excluded. This was achieved without expanding the GTV to 5 mm as proposed by the EORTC guideline [16], questioning this approach when using optimal imaging and a target volume concept with a 60 Gy volume for subclinical disease in LAHNSCC.

As limitations of our study, we acknowledge that the low patient number and therefore limited numbers of diverse tumor locations and stages as well as the fact that the delineation was done by only one physician might imply a certain likelihood of chance findings. Yet, since there is a large interobserver heterogeneity in delineating the primary tumor based on MRI [17], using additional observers might not add accuracy.

We did not use hybrid imaging like PET/MRI, which might be the optimal method, because this technology is still limited to specialized centers [18].

We conclude that MRI, especially in RT treatment position, improves target volume definition in LAHNSCC patients. In this respect, a general challenge in future will be the training of radiation oncologists in MRI-based delineation of LAHNSCC as significant differences in target volume delineation were reported even amongst experienced radiation oncologists [17].

## Conclusions

The usage of additional dedicated MRI imaging for definition of the primary tumor in LAHNSCC results in relevant changes of the GTV and achieves a local control rate of more than 90%.

## Data Availability

The datasets analysed during the current study are available from the corresponding author on reasonable request.
